# A new innovative imaging modality: “extended reality” in endoscopic ultrasonography

**DOI:** 10.1055/a-2512-5145

**Published:** 2025-01-31

**Authors:** Yuki Ishikawa-Kakiya, Hirotsugu Maruyama, Kojiro Tanoue, Akira Higashimori, Shusei Fukunaga, Yasuhiro Fujiwara

**Affiliations:** 1 12936 Department of Gastroenterology, Osaka Metropolitan University, Osaka, Japan

Endoscopic ultrasonography (EUS) is essential for management of pancreaticobiliary diseases; it involves operating the endoscope, interpreting the EUS images, comparing them with other images, and considering needle biopsy or surgery. In addition, the anatomy of each patient must be considered; however, attaining an accurate understanding is a major challenge for trainees.

Extended reality (XR) technology is increasingly being used in medical settings 1 2 3 4 . It allows users to grasp and view a three-dimensional (3D) image from various angles, which enables a sensory understanding in conjunction with endoscopic imaging. We believe that XR support can significantly enhance EUS comprehension and training. We here report a case in which EUS was performed with XR navigation support.

A 67-year-old man underwent EUS for suspicion of a serous cystic neoplasm ( Fig. 1 ). 3D models were constructed through contrast-enhanced computed tomography (CT) and magnetic resonance cholangiopancreatography (MRCP) using SYNAPSE VINCENT (Fujifilm Medical Co., Ltd., Tokyo, Japan). The models were uploaded into the Holoeyes MD system (Holoeyes Inc., Tokyo, Japan) and visualized as 3D holograms in real space through the see-through “HoloLens 2” goggles (Microsoft Corp., Redmond, Washington, USA), allowing simultaneous viewing of the ultrasound monitor and 3D hologram ( Fig. 2 ; Video 1 ). Changing the angle of the 3D image to match that of the EUS image made understanding the depictions of the organs easier. Furthermore, trainees and experts could view the same 3D hologram simultaneously in a virtual session, which proved effective for teaching and reviewing ( Fig. 3 ). By adding a hologram of the endoscope, understanding its movement using the goggles became possible ( Fig. 4 ).

**Fig. 1:**
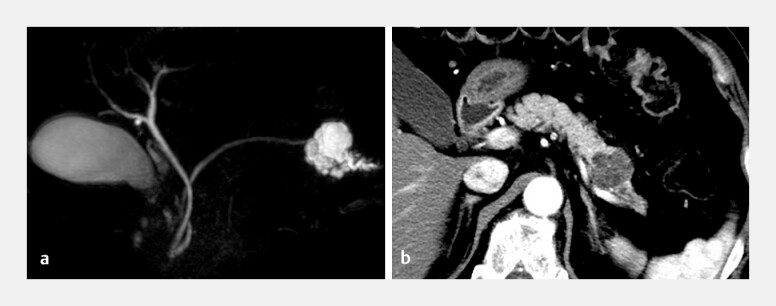
Imaging of a suspected serous cystic neoplasm in the tail of the pancreas on: **a** magnetic resonance cholangiopancreatography; **b** contrast-enhanced computed tomography.

**Fig. 2:**
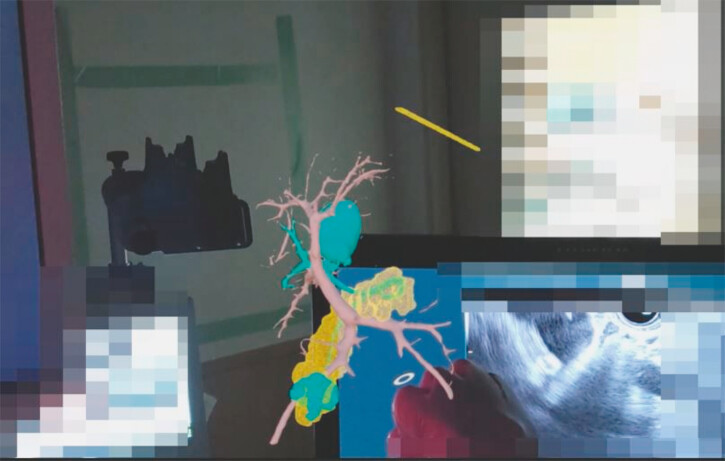
Simultaneous display of the endoscopic ultrasonography image and a 3D hologram through the see-through goggles.

**Fig. 3:**
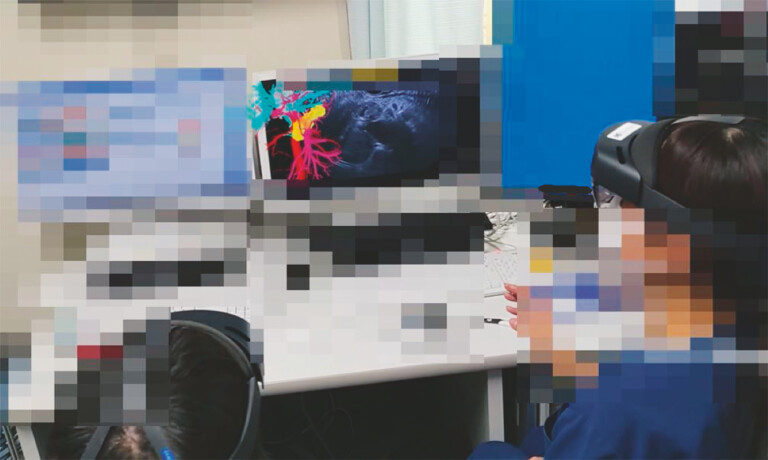
Use of the 3D hologram for review and educational purposes.

**Fig. 4:**
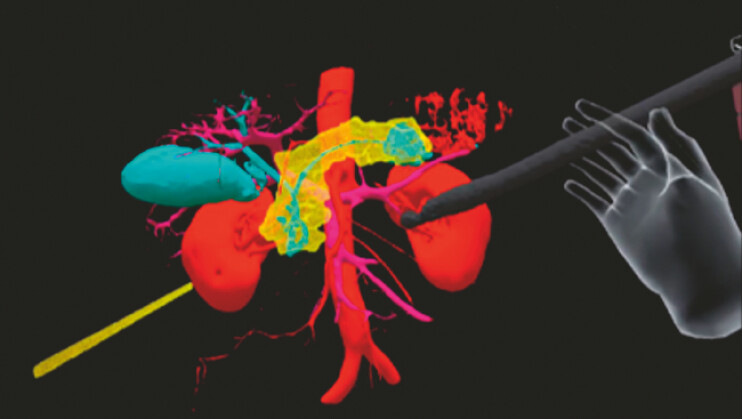
With the immersive goggles, various simulations can be experienced using a 3D hologram.

Extended reality navigation during endoscopic ultrasonography.Video 1

We performed EUS safely with the XR navigation system, allowing for an intuitive understanding of the 3D structure of the organs. This system was also effective for teaching and review purposes. The new method supported by XR could be a breakthrough in this field.

Endoscopy_UCTN_Code_TTT_1AS_2AD

## Notes

Endoscopy E-Videos https://eref.thieme.de/e-videos
*E-Videos* is an open access online section of the journal *Endoscopy* , reporting on interesting cases and new techniques in gastroenterological endoscopy. All papers include a high-quality video and are published with a Creative Commons CC-BY license. Endoscopy E-Videos qualify for HINARI discounts and waivers and eligibility is automatically checked during the submission process. We grant 100% waivers to articles whose corresponding authors are based in Group A countries and 50% waivers to those who are based in Group B countries as classified by Research4Life (see: https://www.research4life.org/access/eligibility/ ). This section has its own submission website at https://mc.manuscriptcentral.com/e-videos .
